# Intra-abdominal fat accumulation is a hypertension risk factor in young adulthood: A cross-sectional study

**DOI:** 10.1097/MD.0000000000005361

**Published:** 2016-11-11

**Authors:** Atsushi Takeoka, Jun Tayama, Hironori Yamasaki, Masakazu Kobayashi, Sayaka Ogawa, Tatsuo Saigo, Hiroaki Kawano, Norio Abiru, Masaki Hayashida, Takahiro Maeda, Susumu Shirabe

**Affiliations:** a Unit of Preventive Medicine, Nagasaki University Graduate School of Biomedical Sciences, Nagasaki, Japan; b Graduate School of Education, Nagasaki University, Nagasaki, Japan; c Department of Endocrinology and Metabolism, Sasebo City General Hospital, Sasebo, Japan; d Center for Health and Community Medicine, Nagasaki University, Nagasaki, Japan; e Department of Cardiovascular Medicine, Nagasaki University Graduate School of Biomedical Sciences, Nagasaki, Japan; f Department of Endocrinology and Metabolism, Nagasaki University Graduate School of Biomedical Sciences, Nagasaki, Japan; g Unit of Community Medicine, Nagasaki University Graduate School of Biomedical Sciences, Nagasaki University, Nagasaki, Japan.

**Keywords:** body fat distribution, electric impedance, intra-abdominal fat, sex characteristics, young adult

## Abstract

Accumulation of intra-abdominal fat is related to hypertension. Despite this, a relationship between hypertension and intra-abdominal fat in young adulthood is not clear. In this study, we verify whether intra-abdominal fat accumulation increases a hypertension risk in young adult subjects.

In a cross-sectional study, intra-abdominal fat area was measured using a dual bioelectrical impedance analysis instrument in 697 university students (20.3 ± 0.7 years, 425 men). Blood pressure and anthropometric factors were measured. Lifestyle variables including smoking, drinking, physical activity, and eating behavior were assessed with questionnaire. High blood pressure risk (systolic blood pressure ≥130 mm Hg and/or diastolic blood pressure ≥85 mm Hg) with increasing intra-abdominal fat area was evaluated.

Participants were divided into 5 groups according to their intra-abdominal fat area (≤24.9, 25–49.9, 50–74.9, 75–99.9, and ≥100 cm^2^). As compared with the values of the smallest intra-abdominal fat area group, the crude and lifestyle-adjusted odds ratios (ORs) were elevated in larger intra-abdominal fat area groups [OR 1.31, 95% confidence interval (CI) 0.66–2.80; OR 3.38, 95% CI 1.60–7.57; OR 7.71, 95% CI 2.75–22.22; OR 18.74, 95% CI 3.93–105.64, respectively). The risk increase was observed only in men.

Intra-abdominal fat accumulation is related to high blood pressure in men around 20 years of age. These results indicate the importance of evaluation and reduction of intra-abdominal fat to prevent hypertension.

## 1 Introduction

Hypertension is a major risk factor for cardiovascular diseases, and the main risk factor for hypertension is obesity. In a review from the Framingham Heart Study,^[1]^ in which participants were prospectively followed for up to 44 years, it was estimated that excess body weight (including overweight and obesity) accounted for approximately 26% and 28% of hypertension cases in men and women, respectively, and for approximately 23% and 15% of coronary heart disease cases in men and women, respectively.

The mechanisms of hypertension seen with obesity are explained by hemodynamic alterations (eg, elevation in cardiac output and relatively elevated systemic vascular resistance) and abnormalities in lipid and glucose metabolism related to fat distribution.^[2]^ In particular, the risk is greatest in patients with abdominal obesity. In several studies, intra-abdominal fat (IAF) accumulation is positively related to the increasing prevalence of hypertension.^[3,4]^

The standard methods to evaluate IAF area (IAFA) are computed tomography (CT) and magnetic resonance imaging (MRI).^[5]^ However, because of the high cost and radiation exposure, these imaging studies are not always feasible. The dual bioelectrical impedance analysis (dual BIA) instrument was recently developed.^[6]^ It can determine IAFA by measuring truncal impedance and surface impedance at the abdomen separately, each of which reflects the truncal adiposity and the subcutaneous adiposity, respectively. The dual BIA instrument is optimized for use in a wide range of human variation by analyzing the size of the effect that each parameter such as age and sex can have on the calculation outcomes utilizing information technology. The IAFA measured with the dual BIA instrument has good correlation with CT-measured IAFA.^[7–9]^

The prevalence of obesity increases mostly in young adulthood around 20 years of age.^[10]^ Once a person becomes obese, it is difficult to reduce and maintain the body weight.^[11]^ Therefore, this time period is important for intervention.^[12]^ The reduction of IAF is thought to be beneficial for preventing hypertension, but the relationship between high blood pressure (BP) and IAFA in young adulthood is not clear. In this study, we verify whether IAF accumulation increases the risk of high BP in young adult Japanese subjects.

## 2 Methods

### 2.1 Study design and participants

**Figure 1:**
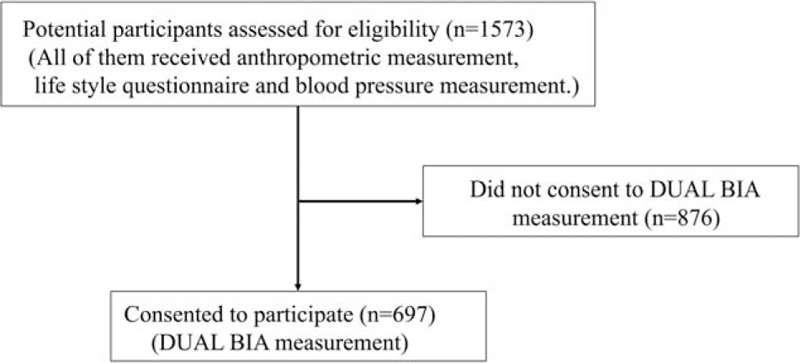
Study flow chart. Of 1573 subjects, 876 did not provide consent for participation. We analyzed the data of the remaining 697 participants who underwent dual bioelectrical impedance analysis (BIA) measurement.

This cross-sectional study included 1573 young adults (907 men and 666 women). All the participants were third-grade students of Nagasaki University. They underwent annual health checkups, including anthropometric measurements, a self-reporting lifestyle questionnaire, and BP measurement from April to June 2013. None of them took antihypertensive medication. Informed consent was obtained for undergoing IAFA measurement with the dual BIA instrument (Omron Dual scan HDS-2000; Omron, Kyoto, Japan). Then, 697 (425 men and 272 women) agreed to undergo abdominal fat composition measurement and the data were used for analysis (Fig. 1). Comparing 876 subjects who did not provide consent for participation, 697 participants were heavier and had higher body mass index (BMI) and waist circumference (WC) (body weight 56.8 ± 8.2 vs 60.0 ± 11.7 kg; *P* < 0.0001; BMI 20.6 ± 1.9 vs 21.6 ± 3.3 kg/m^2^; *P* < 0.0001; WC 73.2 ± 5.4 vs 76.2 ± 9.2 cm; *P* < 0.0001).

### 2.2 Anthropometric measurements

Anthropometric measurements including height, body weight, and WCs were assessed. Each participant was asked to wear light clothing and no shoes during the measurements. Height and body weight were measured to the nearest 0.1 cm and 0.1 kg, respectively, using an electronic scale with attached stadiometer (TANITA DC-250; Tanita Inc., Tokyo, Japan). WC was measured at the midpoint between the lower costal margin and the level of the anterior superior iliac crest using a nonelastic tape.

### 2.3 Blood pressure measurement

Blood pressure was measured serially with an electrical sphygmomanometer (DM-3000; Japan Precision Instruments Inc., Gunma, Japan). Participants were asked to rest quietly for over 5 minutes before BP measurement. The representative values were calculated as follows. BP measurements were performed twice with the participant in the sitting position. The measurement with lower systolic BP (SBP) was chosen as the representative value. In this study, “high BP” was defined as SBP ≥130 mm Hg and/or diastolic BP (DBP) ≥85 mm Hg.

### 2.4 Dual BIA method and instrumentation

The dual BIA instrument calculates the cross-sectional area of IAF at the level of the umbilicus based on the measurement of electrical potentials resulting from the application of small electrical currents in the 2 different body compartments. The principles of IAFA determination by the dual BIA instrument were described previously.^[13,14]^ Briefly, the dual BIA instrument consists of a bioelectric impedance component that measures truncal and surface impedance of the body, and a device that measures the physical size of the abdomen. The 2 sets of electrodes are for limb and truncal placement. The limb electrodes consist of 4 clip-on electrodes placed on the wrists and ankles. The truncal electrodes are 8 pairs of electrodes 6 cm apart longitudinally that are fixed to a belt in pairs, 1 each for front and back, and positioned at an equal distance. The belt is adjustable, allowing the electrodes to be centered on the mid-sagittal line at the level of the umbilicus in the supine position. The truncal impedance is measured by applying electrical currents between the upper and lower limb leads and reading the voltage from the electrodes around the abdominal circumference. The surface impedance is measured by applying and measuring the voltage from the abdominal circumference electrodes. Other details of the study method were described previously.^[6,7,13,14]^ Measurements were performed at the end of expiration phase in normal breathing because measurement is affected by breathing phase. To avoid the interference of hydration status on results, measurements were done at the fasting morning with minimum of drink.

### 2.5 Lifestyle assessment

We assessed participants’ smoking status, drinking status, physical activity, and eating behavior with a self-reporting questionnaire. Current smoker was defined as smoking more than 1 cigarette per day habitually. Current drinker was defined as drinking more than 1 alcohol drink per week habitually. Physical activity was determined by asking participants whether they belong to sports clubs and/or if they walk more than 30 minutes per day. Eating behaviors were assessed using the 30 questions of the 50 items on the Sakata Eating Behavior Questionnaire,^[15,16]^ which have been shown to reliably discriminate between obesity patients and healthy people. The 30 questions were classified into 7 categories: cognition of constitution (3 items), motivation for eating (3 items), substitute eating and drinking (6 items), feeling of satiety (5 items), eating style (3 items), meal contents (5 items), and eating rhythm abnormalities (5 items). The questionnaire was completed on a 4-point Likert scale ranging from “strongly disagree,” “somewhat disagree,” and “somewhat agree,” to “strongly agree,” with higher scores indicating eating behaviors that lead to obesity.

### 2.6 Statistical analysis

All clinical data were summarized as means and SDs. An unpaired *t* test or chi-square test was used to compare groups. To evaluate the relationship between IAFA and high BP, the participants were divided into 5 groups according to their IAFA (≤24.9, 25–49.9, 50–74.9, 75–99.9, and ≥100 cm^2^). Then, crude odds ratios (ORs) of high BP and 95% confidence intervals (95% CIs) compared with the smallest IAFA group (≤24.9 cm^2^) as reference were calculated. Multivariate logistic regression was then conducted by using smoking status, drinking status, physical activity, and eating behavior with the scores of the self-reporting questionnaire as the variables. Sex difference was also evaluated. The significance threshold was set at *P* < 0.05. JMP ver. 10.0 software (SAS Institute, Cary, NC) was used for all statistical analyses.

### 2.7 Ethical issues

The investigational protocol was approved by the research ethics committee of Nagasaki University, and informed consent was obtained from all the participants.

## 3 Results

Table 1 shows the clinical characteristics of the participants in the present study. High BP was found in 88 of 697 participants, most of whom were men (74/88, 84.1%). The prevalence of high BP in men was higher than that in women (74/425, 17.4% vs 14/272, 5.1%; *P* < 0.0001). As compared with the normotensive group, significant increases in height, weight, BMI, and WC were seen in the high BP group (165.7 ± 8.6 vs 170.9 ± 7.7 cm; *P* < 0.0001; 58.3 ± 10.2 vs 72.1 ± 14.2 kg; *P* < 0.0001; 21.2 ± 2.9 vs 24.6 ± 4.3 kg/m^2^; *P* < 0.0001; and 75.1 ± 8.1 vs 84.1 ± 12.0 cm; *P* < 0.0001, respectively). The prevalence of current smokers and current drinkers were not significantly different between both groups (*P* = 0.24 and *P* = 0.32 respectively). Physical activity and the scores of eating-behavior questionnaire were not significantly different between both groups (*P* = 0.24 and *P* = 0.39 respectively). IAFA and subcutaneous fat area (SFA) in the high BP group were significantly larger than those in the normotensive group (53.9 ± 28.8 vs 36.3 ± 17.4 cm^2^; *P* < 0.0001 and 176.2 ± 92.8 vs 116.2 ± 64.2 cm^2^; *P* < 0.0001, respectively).

**Table 1:**
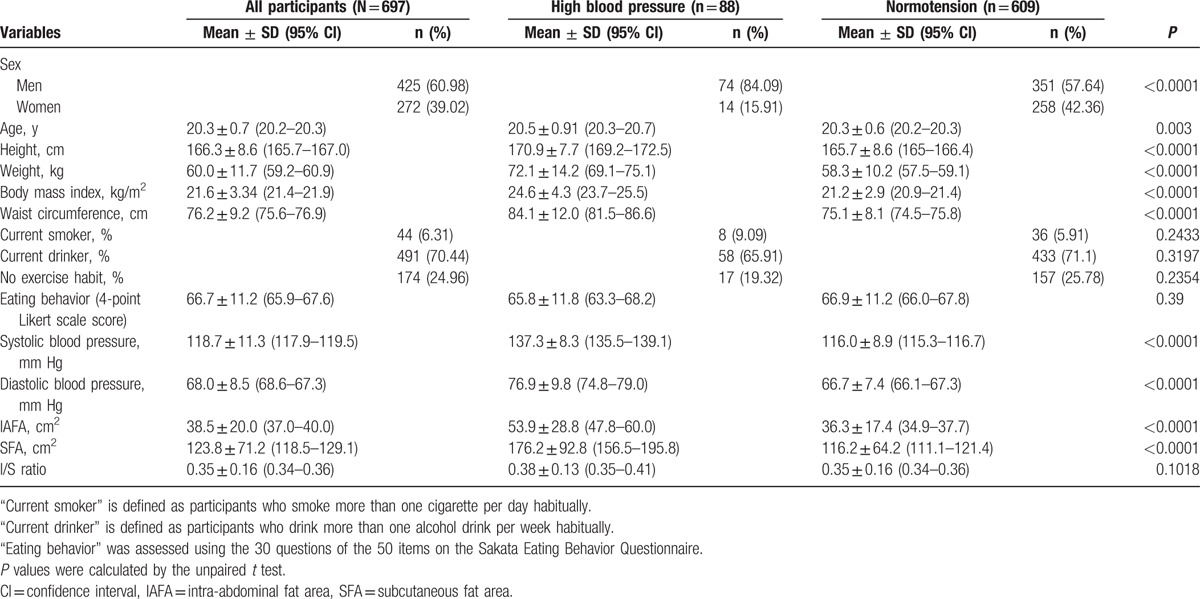
Demographic data of high blood pressure and normotension groups.

To evaluate the relationship between IAFA and high BP, the participants were divided into 5 groups according their IAFA, and were designated as quintile 1 through 5. As compared with the values of the smallest IAFA group (quintile 1), the crude ORs and 95% CIs were elevated in quintiles 2, 3, 4, and 5 (Table 2). After adjusting for current smoking, current drinking, physical activity, and scores of the eating-behavior questionnaire, the significance of ORs were preserved in the larger IAFA groups such as quintiles 3, 4, and 5.

**Table 2:**
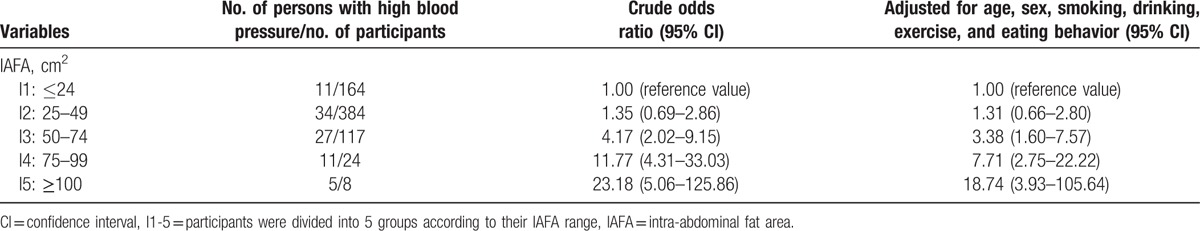
Relationship between IAFA and risk of high blood pressure in all participants.

The relationship between IAFA and risk of high BP in men and women is shown in Table 3. In men, using the values of the smallest IAFA group (quintile 1) as reference, crude ORs and 95% CIs of high BP showed an increase in the risk of high BP in quintiles 2, 3, 4, and 5. After adjusting for current smoking, current drinking, physical activity, and scores of the eating-behavior questionnaire, the significance of the ORs were preserved in the larger IAFA groups such as quintiles 3, 4, and 5. In women, the association of IAFA with high BP was, however, not significant, even when comparing the highest quintile and lowest quintile of IAFA (data not shown). The correlation coefficient (CC) between IAFA and BP in both sexes was analyzed (Table 4). The CCs between IAFA and SBP or DBP were significant in men (*r* = 0.35, *P* < 0.0001; and *r* = 0.22, *P* < 0.0001, respectively); however, in women, no significant correlations were found (*r* = 0.05, *P* = 0.40; and *r* = 0.06, *P* = 0.33, respectively).

**Table 3:**
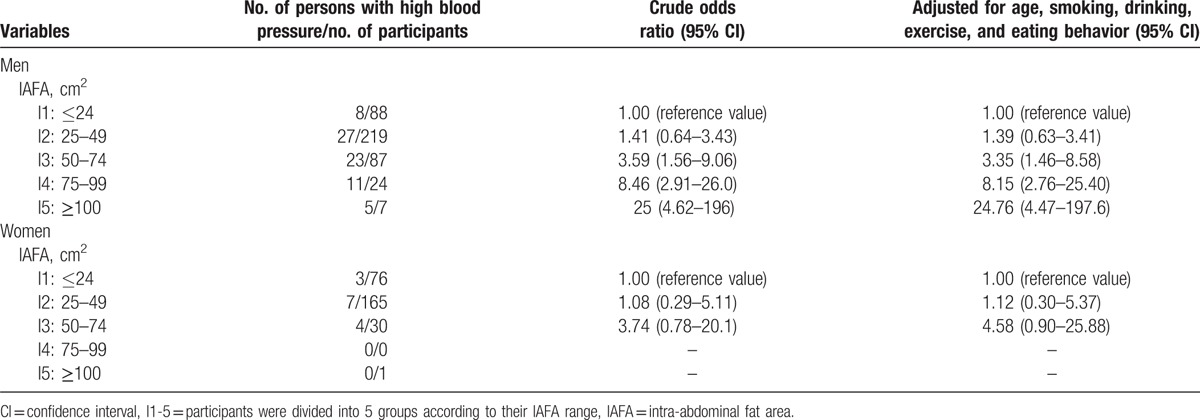
Relationship between IAFA and risk of high blood pressure in men and women.

**Table 4:**
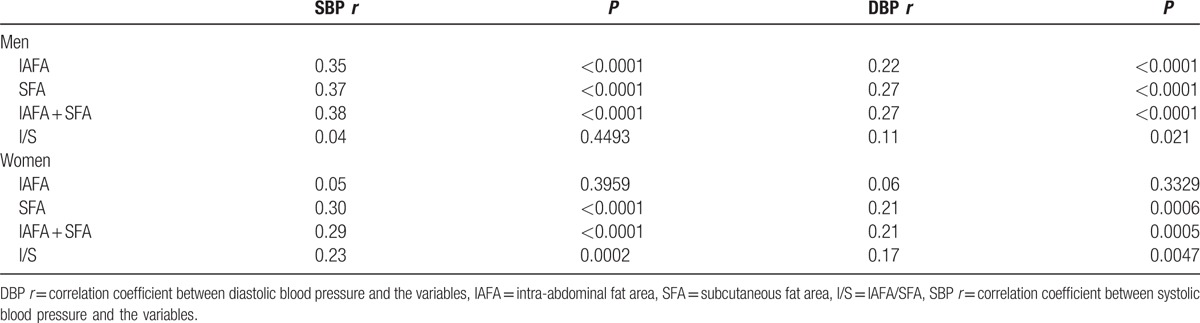
Sex differences of the correlation coefficient between the body fat composition and blood pressure.

## 4 Discussion

In the present study, we hypothesized that IAF accumulation increases the risk of high BP. The results showed a significantly increased risk of high BP associated with IAF accumulation in men. This relationship was significant when comparing between groups whose IAFA was ≤100 cm^2^. On the contrary, the relationship was not significant in women. Therefore, our hypothesis was partially proved in men. Most of the studies evaluating the relationship between IAF accumulation and risk of hypertension are focused on the middle-aged or the older population. To the best of our knowledge, this is the first study that evaluates the risk ratio of high BP according to IAF accumulation in young adults.

Several studies demonstrated the relationship between IAFA and hypertension. Hayashi et al^[3]^ followed up 300 Japanese Americans for about 10 years and reported that IAFA measured by CT at baseline was correlated with increased prevalence of hypertension in the follow-up period. The increased hypertension risk associated with IAFA was significant after adjusting for WC or SFA. Similar results were observed in studies of Japanese subjects^[17]^ and other ethnic groups.^[4]^ The participants of these studies were middle-aged. Our findings showed the risk in young adulthood. One study of adolescents^[18]^ reported a positive relationship between MRI-measured IAFA and BP in boys. Thus, IAF accumulation is associated with high BP at least since adolescence. Although the Japan Society for the Study of Obesity made a caution on overaccumulation of visceral fat as CT-measured IAFA ≥100 cm^2^,^[19]^ in our study, an increased risk of high BP with IAF accumulation was observed in subjects with IAFA ≤100 cm^2^. This result was in line with the previous study that showed CT-measured IAFA was risk of high BP in middle age even in subjects with BMI <25 kg/m^2^ and IAFA ≤100 cm^2^.^[17]^ Therefore, the present study showed that the risk increases with a relatively smaller IAF accumulation than we expected.

The mechanisms of hypertension associated with IAF are not fully understood. Mainly 2 mechanisms are conceived. One is activation of the renin-angiotensin system (RAS) by adipose tissue. Adipose tissue produces RAS-associated proteins including angiotensinogen, angiotensin-converting enzyme (ACE), prorenin receptor, cathepsin D, and tonin.^[20]^The expression of the angiotensinogen gene is more predominant in IAF than subcutaneous fat.^[21]^ Yiannikouris et al^[22]^ compared the adipocyte angiotensinogen-deficient mice with wild-type mice to investigate whether high fat-feeding induces RAS activation and BP elevation. Adipocyte angiotensinogen deficiency prevented high fat-induced elevations in plasma angiotensin II concentrations and SBP. This result suggests that adipose tissue serves as a major source of angiotensin II in the development of obesity-related hypertension.^[22]^ Another mechanism is sympathoexcitability associated with fat accumulation. Leptin is one of the adipokines, and the blood concentration of leptin reflects the amount of adipose tissue.^[23]^ Leptin is a critical contributor to obesity-related hypertension through increasing of sympathetic nerve activity.^[24]^ Other than these mechanisms, insulin resistance, other adipokines, and inflammatory response associated with IAF accumulation are thought to be involved.^[25]^

Pausova et al^[18]^ pointed out the sex difference in the relationship between IAF accumulation and BP. In the present study, unlike in men, no significant association between IAFA and BP was observed in women. On the contrary, in studies in the middle-aged or the older population, especially in postmenopausal women, such sex difference is not observed.^[26,27]^ These findings indicate sex hormone effects. Estrogens increase systemic blood concentration of angiotensinogen,^[28]^ but reduce the activation of ACE,^[29]^ down-regulate AT1 receptor,^[30]^ and up-regulate AT2 receptor.^[31]^ Taken together, estrogens inhibit RAS activity. This effect of estrogen may counteract the IAF-associated activation of RAS. Additionally, estrogen is supposed to have effect on leptin-induced sympathoexcitability.^[32]^ Androgens also have an effect on IAF-associated hypertension. A functional variation in the androgen-receptor gene in boys showed a difference in BP and sympathetic modulation of the vasomotor tone.^[33]^ In large cohort studies, the prevalence of hypertension is lower in premenopausal women, but the prevalence gradually increases and becomes equal to that of men in the postmenopausal period.^[34]^ Thus, IAF accumulation is thought to have an effect on BP according to sex. Further investigation about the relationship between sex hormones and IAF-associated hypertension is required.

Our results suggest that reducing IAF in young may be beneficial. But “when and how the intervention should be” is controversial. Young adulthood around 20 years of age might be a reasonable period for intervention. Around the age of 20, defined by Arnett as “Emerging Adulthood,”^[35]^ is an important period for making lifestyle changes. In industrialized countries, most of young adults about this age leave their families and begin to make their own decisions. The decisions include those related to eating behavior, physical activities, smoking, and drinking, among others.^[9]^ In fact, a large portion of the population becomes obese in these years.^[7,8]^

A systematic review evaluated the relationship between weight change and BP difference in adults.^[36]^ The relationship found between weight and SBP difference was 1 kg:1 mm Hg. However, in obese subjects, it is difficult to reduce weight and maintain it because weight reduction induces increased appetite and decrease of energy expenditure.^[37]^ This phenomenon is caused by cross-talk between adipose tissue and the central nervous system with adipokines such as leptin and ghrelin.^[38]^ Thus, an effective weight-reduction program should take into account these mechanisms. Many studies have investigated the efficacy of intervention in obesity-related hypertension. However, few studies have evaluated changes in IAF.^[39]^ The reason for this is the difficulty to measure IAF. Although the gold standards for measuring IAF are CT or MRI, these are not suitable for frequent evaluation because of the costs and radiation exposure. In the present study, we used the dual scan. This device is noninvasive, relatively costless, and easy to manipulate. Therefore, this device facilitates the frequent evaluation of IAF.^[12]^ The precise evaluation of the relationship between IAF change and BP will add new knowledge about appropriate intervention.

This single-center cross-sectional study of Japanese young adults has mainly 4 limitations. First, because the sample size of female subjects was small, sex-stratified multiple logistic analysis (Table 3) failed to show the relationship between IAFA and high BP clearly. We also compared the tertiles of IAFA in women. After adjustment of age, smoking, exercise, and eating behavior, the risk of high BP was not different within the groups (using the value of the lower IAFA trtile group as reference, ORs and 95% CIs of high BP of middle and higher tertile groups were OR 0.96, 95% CI 0.11–8.17 and OR 0.58, 95% CI 0.07–3.59, respectively). In addition, liner regression analysis showed no significant relationship between IAFA and BP in women (Table 4). Second, although family history is an important factor of high BP, variables in this study do not include family history. However, in 561 of 697 participants, family histories were confirmed, and after adjusting for family history, the results were not different. Third, salt sensitivity is supposed to be involved in high BP with IAF accumulation. Therefore, the amount of salt intake can be a bias, but these data were not obtained in the present study. In general, young adults with normal renal function have sufficient sodium extraction abilities. None of the participants had a history of kidney disease or detectable proteinuria. Fourth, this is a cross-sectional study, and it cannot demonstrate a causal relationship. However, the present study results suggest that IAF accumulation is a critical risk factor for high BP and an important target for intervention. Further studies are needed to elucidate the relationship between IAF change and BP.

## 5 Conclusions

Young adulthood around 20 years of age is an important time period for preventing hypertension. This study revealed that IAF accumulation is related to high BP in Japanese men around 20 years of age. Furthermore, an increase in the risk of high BP was observed in subjects with IAFA ≤100 cm^2^. These results indicate the importance of evaluation and reduction of IAF to prevent hypertension.
